# Supplementary material for: Predictors of Clavien–Dindo Grade III–IV or Grade V Complications after Metastatic Spinal Tumor Surgery: An Analysis of Sociodemographic, Socioeconomic, Clinical, Oncologic, and Operative Parameters
Source: Cancers (Basel). 2024 Aug 1;16(15):2741. doi: 10.3390/cancers16152741 (PMC11311255; doi:10.3390/cancers16152741)
Supplement: Supplementary file 1 [file cancers-16-02741-s001.zip › cancers-3063919-supplementary.pdf]

Predictors of Clavien-Dindo Grade III-IV or Grade V Complications after Metastatic Spinal Tumor Surgery: An Analysis of Sociodemographic, Socioeconomic, Clinical, Oncologic, and Operative Parameters

Rafael De la Garza Ramos MD [1,2*], Jessica Ryvlin BS [1], Ali Haider Bangash MBBS [1], Mousa K Hamad [1,2], Mitchell S Fourman MD Phil[1,3], John H Shin MD[4], Yaroslav Gelfand MD [1,2], Saikiran Murthy DO [1,2] and Reza Yassari MD [1,2,]

1 Spine Research Group, Montefiore Medical Center/Albert Einstein College of Medicine, Bronx, 10467 NY, USA; rafdelag@gmail.com (R.D.L.G.R.); jessica.ryvlin@einsteinmed.org (J.R.); alihaider2022-010@stmu.edu.pk (A.H.B.); hamadmousa@gmail.com (M.K.H.); ygelfand@montefiore.org (Y.G.); samurthy@montefiore.org (S.M.); ryassari@montefiore.org (R.Y.)

2 Department of Neurological Surgery, Montefiore Medical Center Albert Einstein College of Medicine, Bronx, 10467 NY, USA; e-mail@e-mail.com

3 Department of Orthopaedic Surgery, Montefiore Medical Center Albert Einstein College of Medicine, Bronx, 10467 NY, USA; mfourman@montefiore.org (M.S.F.)

4 Department of Neurological Surgery, Massachusetts General Hospital, Harvard Medical School, Boston, 02115 MA, USA; Shin.John@mgh.harvard.edu (J.H.S.)

* Correspondence: rdelag@montefiore.org

Supplementary Materials:

Table S1. Eastern Cooperative Oncology Group (ECOG) Performance Status Scale[1]

Grade	Description
0	Fully active, able to carry on all pre-disease performance without restriction
1	Restricted in physically strenuous activity but ambulatory and able to carry out work of a light or sedentary nature (e.g., light house work, office work)
2	Ambulatory and capable of all self-care but unable to carry out any work

	activities; up and about more than 50% of waking hours
3	Capable of only limited self-care; confined to bed or chair more than 50% of waking hours
4	Completely disabled; cannot carry on any self-care; totally confined to bed or chair
5	Dead

Table S2. American Society of Anesthesiologists (ASA) Physical Status Classification System[2]

ASA Class	Definition
ASA I	A normal healthy patient
ASA II	A patient with mild systemic disease
ASA III	A patient with severe systemic disease
ASA IV	A patient with severe systemic disease that is a constant threat to life
ASA V	A moribund patient who is not expected to survive without the operation
ASA VI	A declared brain-dead patient whose organs are being removed for donor purposes

Note: "E" is added to the classification in case of emergency surgery (e.g., ASA III-E).

Table S3. Frankel Classification of Spinal Cord Injury[3]

Grade	Description
A	Complete paralysis: No motor or sensory function below the level of injury
B	Sensory only: No motor function, but some sensory function below the level of injury
C	Motor useless: Some motor function below the level of injury, but not functional
D	Motor useful: Functional motor ability below the level of injury
E	Recovery: Normal motor and sensory function

Table S4. Modified Bauer Score for Prognostic Assessment of Spinal Metastases[4]

Prognostic Factor	Score
No visceral metastases	1
No lung cancer	1
Primary tumor: breast, kidney, lymphoma, or myeloma	1
Solitary skeletal metastasis	1
Interpretation	
**Total Score**	**Prognosis**
0-1	Poor
2	Intermediate
3-4	Good

Table S5. Spinal Instability Neoplastic Score (SINS)[5]

Component	Score
**Location**	
- Junctional (occiput-C2, C7-T2, T11-L1, L5-S1)	3
- Mobile spine (C3-C6, L2-L4)	2
- Semi-rigid (T3-T10)	1
- Rigid (S2-S5)	0
**Pain**	
- Yes	3
- Occasional pain but not mechanical	1
- Pain-free lesion	0
**Bone lesion**	
- Lytic	2
- Mixed (lytic/blastic)	1
- Blastic	0
**Radiographic spinal alignment**	
- Subluxation/translation present	4
- De novo deformity (kyphosis/scoliosis)	2
- Normal alignment	0
**Vertebral body collapse**	
- >50% collapse	3
- <50% collapse	2
- No collapse with >50% body involved	1
- None of the above	0
**Posterolateral involvement of spinal elements**	
- Bilateral	3
- Unilateral	1
- None of the above	0
**Interpretation**	
**Total Score**	**Spinal Stability**
0-6	Stable
7-12	Potentially unstable
13-18	Unstable

References

1. Oken, M.M.; Creech, R.H.; Tormey, D.C.; Horton, J.; Davis, T.E.; McFadden, E.T.; Carbone, P.P. Toxicity and Response Criteria of the Eastern Cooperative Oncology Group. *Am. J. Clin. Oncol.* **1982**, *5*, 649–655.
2. Doyle, D.J.; Hendrix, J.M.; Garmon, E.H. American Society of Anesthesiologists Classification. In *StatPearls*; StatPearls Publishing: Treasure Island (FL), 2024.
3. Frankel, H.L.; Hancock, D.O.; Hyslop, G.; Melzak, J.; Michaelis, L.S.; Ungar, G.H.; Vernon, J.D.; Walsh, J.J. The Value of Postural Reduction in the Initial Management of Closed Injuries of the Spine with Paraplegia and Tetraplegia. I. *Paraplegia* **1969**, *7*, 179–192, doi:10.1038/sc.1969.30.
4. Leithner, A.; Radl, R.; Gruber, G.; Hochegger, M.; Leithner, K.; Welkerling, H.; Rehak, P.; Windhager, R. Predictive Value of Seven Preoperative Prognostic Scoring Systems for Spinal Metastases. *Eur. Spine J. Off. Publ. Eur. Spine Soc. Eur. Spinal Deform. Soc. Eur. Sect. Cerv. Spine Res. Soc.* **2008**, *17*, 1488–1495, doi:10.1007/s00586-008-0763-1.
5. Fisher, C.G.; DiPaola, C.P.; Ryken, T.C.; Bilsky, M.H.; Shaffrey, C.I.; Berven, S.H.; Harrop, J.S.; Fehlings, M.G.; Boriani, S.; Chou, D.; et al. A Novel Classification System for Spinal Instability in Neoplastic Disease: An Evidence-Based Approach and Expert Consensus from the Spine Oncology Study Group. *Spine* **2010**, *35*, E1221-1229, doi:10.1097/BRS.0b013e3181e16ae2.
